# New Non-Toxic Semi-Synthetic Derivatives from Natural Diterpenes Displaying Anti-Tuberculosis Activity

**DOI:** 10.3390/molecules201018264

**Published:** 2015-10-07

**Authors:** Priscilla M. Matos, Brian Mahoney, Yohan Chan, David P. Day, Mirela M. W. Cabral, Carlos H. G. Martins, Raquel A. Santos, Jairo K. Bastos, Philip C. Bulman Page, Vladimir C. G. Heleno

**Affiliations:** 1 Núcleo de Pesquisas em Ciências Exatas e Tecnológicas, Universidade de Franca, Franca 14404-600, Brazil; E-Mails: pcxpm1@nottingham.ac.uk (P.M.M.); mirela@com4.com.br (M.M.W.C.); carlos.martins@unifran.edu.br (C.H.G.M.); raquel.santos@unifran.edu.br (R.A.S.); 2 School of Chemistry, University of East Anglia, Norwich Research Park, Norwich, NR4 7TJ, UK; E-Mails: mahoneybri@gmail.com (B.M.); y.chan@uea.ac.uk (Y.C.); ddukbr@gmail.com (D.P.D.); p.page@uea.ac.uk (P.C.B.P.); 3 Faculdade de Ciências Farmacêuticas de Ribeirão Preto, Universidade de São Paulo, Ribeirão Preto 14040-903, Brazil; E-Mail: jkbastos@fcfrp.usp.br

**Keywords:** kaurenoic acid, copalic acid, structural modification, diterpene derivatives, anti-tuberculosis activity, cytotoxicity, *Mycobacterium tuberculosis*

## Abstract

We report herein the synthesis of six diterpene derivatives, three of which are new, generated through known organic chemistry reactions that allowed structural modification of the existing natural products kaurenoic acid (**1**) and copalic acid (**2**). The new compounds were fully characterized using high resolution mass spectrometry, infrared spectroscopy, ^1^H- and ^13^C-NMR experiments. We also report the evaluation of the anti-tuberculosis potential for all compounds, which showed some promising results for *Micobacterium tuberculosis* inhibition. Moreover, the toxicity for each of the most active compounds was also assessed.

## 1. Introduction 

Tuberculosis (TB) is a widely spread respiratory disease which caused 1.3 million deaths worldwide in 2012 [1]. It is considered the second leading cause of death from a single infectious agent, after HIV [1]. TB is a bacterial respiratory illness, caused by *Micobacterium tuberculosis* [2]. It is contagious and airborne, which makes it somewhat difficult to avoid or to control. This disease mostly affects the lungs, causing coughing, and later leading to chest pains, weakness, weight loss and fever [2]. In 2012, 8.6 million people contracted TB, among them 530,000 children [1]. Multidrug-resistant TB (MDR-TB) is an even worse public health problem due to bacterial resistance to the available anti-tubercular agents. This leads to a higher rate of death, compared to regular TB. In 2012, 450,000 people developed MDR-TB and 170,000 people died from it in the same year [1], leading to a 37.78% rate of death. In the same period the rate of death for TB was 15.12%. The number of people diagnosed with MDR-TB in 2012 was nearly the double of people diagnosed with it in 2011 [1]. Billions of dollars have been invested and are required to finance anti-TB programs. In 2013, the amount invested was 6 billion dollars, and there is still a gap of 2 billion per year [1]. 

The most important action to eradicate TB is the use of Bacille Calmette-Guerin (BCG) vaccine, the only one used to prevent this disease [3]. The policies for applying the BCG vaccine are different around the world. Some countries chose a universal vaccination program, while others only recommend it for some risk groups [3]. The fact is that this eradication is planned by several countries, but to eliminate TB from any country is considered very difficult due to immigration and global travelling [3]. This considerably enhances the importance of effective anti-tubercular agents. 

Anti-tubercular drugs are classified as first, second and third-line drugs, where second-line indicates a less effective drug or expression of some side effects, and third-line indicates a poorly effective drug [4]. Thus, first-line anti-tubercular drugs are the most effective in use nowadays. These drugs are streptomycin, isoniazid, pyrazinamide, rifampicin and ethambutol [4]. The standard treatment for TB is a six month treatment, involving two months of isoniazid, rifampicin, pyrazinamide and ethambutol in the intensive phase, followed by isoniazid and rifampicin in the continuous phase [4]. When we refer to MDR-TB, we refer to the resistance to first-line anti-tubercular drugs [4]. 

Natural products can be found in a wide plethora of living species, with many of them harnessing the potential to possess biological and therapeutic activity of high importance [5,6]. The ever-increasing demand for new drug molecules that act against current or new illnesses has led to greater interest in research conducted on secondary plant metabolites [7,8]. The isolation of a new compound from its natural source and testing its bioactivity is one of the ways, but we wish to focus our research on another alternative; isolation of an already known biologically active compound and modifying key chemical moieties within the molecule to investigate any changes in its bioactivity. These chemical modifications can be performed through biotransformation, utilizing enzymes, or chemically, with the hope of using cheap and commercially available reagents, as we have reported previously [9,10]. 

**Figure 1:**
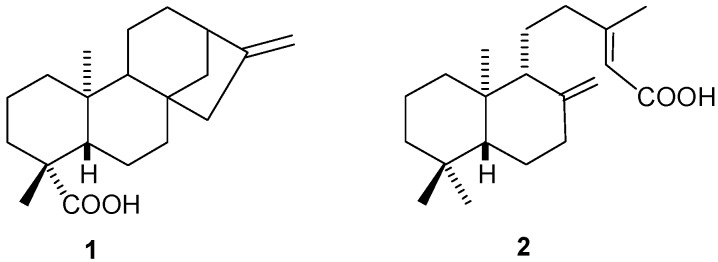
Biologically active diterpenes **1** and **2**.

Diterpenes are a large class of natural compounds that exhibit a range of interesting biological activities such as antimicrobial [11,12], anti-inflammatory [13] gastroprotective [14], anti-spasmodic [15], among others [16]. Due to our research interest on structural modification of terpenoids [17,18] and on the evaluation of biological activities of natural terpenoids and their semisynthetic derivatives [10,12,19], we decided to perform some structural modifications in the natural diterpenes kaurenoic acid (**1**) and copalic acid (**2**) (Figure 1), and to evaluate the variation of the anti-tubercular activity of the different obtained derivatives. This was proposed taking into account the displayed activities of kauranes [16] and labdanes [12], together with the commercial availability of natural sources of kaurenoic acid (**1**) and copalic acid (**2**). Both substances are the major components in each one’s botanical source, kaurenoic acid (**1**) from *Mikania glomerata* (aerial parts) and copalic acid (**2**) from *Copaifera langsdorffii* (oleoresin). Moreover, both compounds have also previously displayed antimicrobial activity [9,11,12].

If the proposed structural modifications were to lead to more active compounds, with no expressive toxicity, we could contribute to the search for anti-TB and anti-MDR-TB agents. The attempt to improve terpenoids activity through structural modification is widely used [20,21] and even inspires some company’s business model [22,23].

We herein present our research results on submitting those natural substances to high-yielding transformations, such as epoxidation, ozonolysis, Baeyer-Villiger and aldol reactions, together with the promising anti-tubercular results obtained. Toxicity was also evaluated for the starting materials and obtained compounds.

## 2. Results and Discussion

### 2.1. Diterpenes

#### 2.1.1. Kaurenoic Acid (**1**)

The certified dried and powdered aerial parts of *Mikania glomerata* were purchased from the Brazilian company called “Nutri Comércio de Ervas LTDA”, based on the city of São Paulo—SP. The extraction was performed as described in experimental section and the obtained kaurenoic acid (**1**) was identified by NMR experiments. This procedure yielded enough **1** to perform the structural modifications and all biological assays.

#### 2.1.2. Copalic Acid (**2**)

The *Copaifera langsdorffii* Desf. oleoresin (OC, Lot 0790310, manufactured 09/2010) was purchased from Apis Flora, a Brazilian herbal company located in the city of Ribeirão Preto—SP, Brazil. The description of the extraction is detailed in the experimental section, and the obtained copalic acid (**2**) was identified by NMR data analysis. In this case also a sufficient quantity of the natural product was obtained.

### 2.2. Synthetic Modifications

**Scheme 1:**
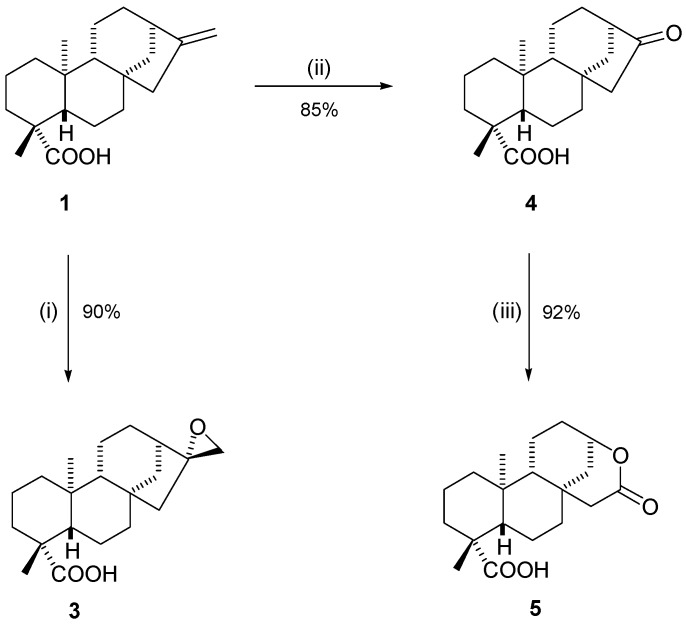
Structural modifications in kaurenoic acid (**1**). *Reagents and Conditions*: (i) **1** (1 equiv.), *m*-CPBA (2 equiv.), CHCl_3_, 0 °C, 24 h; (ii) **1** (1 equiv.), O_3_, CH_2_Cl_2_, −72 °C, 15 min, then (CH_3_)_2_S, −72 °C to r.t.; (iii) **4** (1 equiv.), *m*-CPBA (2 equiv.), r.t., 24 h.

After successful isolation, compounds **1** and **2** were then subjected to a series of chemical reactions aiming to modify the core structure. Six different semisynthetic derivatives were obtained, each containing new key moieties that could be evaluated to detect any increases in activity during MIC tests against tuberculosis cell lines. Looking firstly at the modifications to kaurenoic acid (**1**), we report three successful reactions (Scheme 1). 

The epoxidation of kaurenoic acid **1** had previously been reported in the literature [24,25], and the reaction proceeded in our lab in 90% yield, affording compound **3** as a colorless oil (Scheme 1, step i). Ozonolysis of compound **1** proceeded for 15 min at a reduced temperature to afford the ketone product **4** as a colorless oil in 85% yield (Scheme 1, step ii). Further reaction of ozonolyis product **4** with *m*-CPBA, at room temperature led to the formation of Baeyer-Villiger product **5** in excellent yield (92%, Scheme 1, step iii). Confirmation of each of the products **3**, **4** and **5** was achieved through analysis of the ^1^H- and ^13^C-NMR spectra, along with their IR spectra and further confirmation by high resolution mass spectrometry. DEPT-135 and 2D-NMR experiments were also very useful to confirm assignments. In special, the position of the inserted oxygen atom in the formation of compound **5** was confirmed through the observation of a 1H signal at 4.72 ppm at the ^1^H-NMR spectrum.

**Scheme 2:**
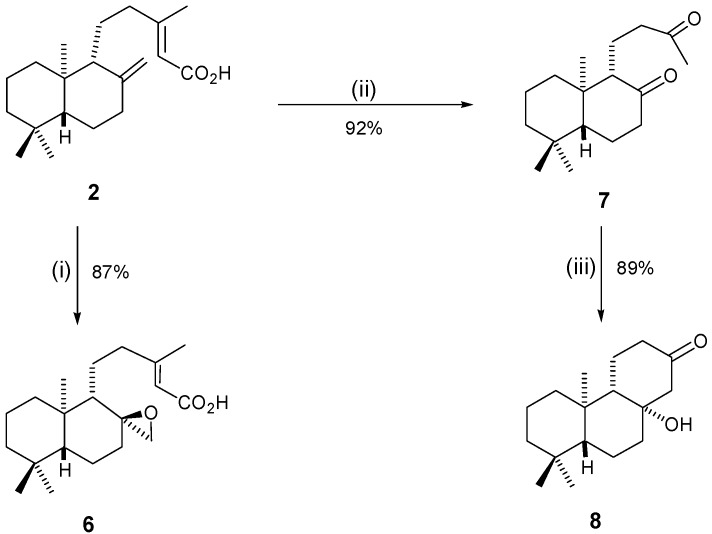
Structural modifications in copalic acid (**2**). *Reagents and Conditions*: (i) **2** (1 equiv.), *m*-CPBA (1.5 equiv.), CHCl_3_, 0 °C, 24 h; (ii) **2** (1 equiv.), O_3_, CH_2_Cl_2_, −72 °C, 15 min, then (CH_3_)_2_S, −72 °C to r.t.; (iii) KO*t*Bu (12 equiv.) in THF, then **7** (1 equiv.) added, r.t., 18 h.

Looking at our second isolated natural product, copalic acid (**2**), we devised a number of structural modifications to this molecule, either using similar reactions to that applied to kaurenoic acid (**1**), or different reactions altogether (Scheme 2). 

All reactions tested on copalic acid were successful, furnishing three new structures: the epoxidation of **2** was very successful, affording the new epoxide **6** in good yield (87%) (Scheme 2, step i) and the ozonolysis of **2** also occurred efficiently, giving the new diketone **7** in a very good yield (92%) (Scheme 2, step ii); aldol conditions applied to compound **7** led to the new derivative **8**, yielding 89% of product (Scheme 2, step iii).

NMR structural assignment, including comparison with the starting material NMR data, led to the confirmation of structures after synthesis. There were performed ^1^H-NMR, ^13^C-NMR {^1^H}, DEPT-135, 2D-NMR experiments. Further confirmation was also achieved by IR spectroscopy and high resolution mass spectrometry. Formation of compound **6** is preferably performed through the insertion of the oxygen from the opposite side of the bulky group at the carbon next to the double bond. This is due to the volume of *m*-CPBA itself. A long range coupling between one of the epoxide hydrogens (W coupling), which is only possible in the proposed isomer, corroborates the proposed stereochemistry. The stereochemistry of the formed ring in compound **8** was assigned on the basis of literature information. Van Wyk and co-workers [26] described exactly the same cyclisation starting from the enantiomer of compound **7**. For the same reasons, the use of compound **7**, led to the obtention of the enantiomer of van Wyk’s product. In addition, our NMR data are also in agreement with the structure [27].

### 2.3. Biological Assays

The evaluation of the anti-tubercular potential performed in this work was conducted against the cell line *Mycobacterium tuberculosis* H37Rv, ATCC 27294, in accordance with a strict experimental procedure, as described in the experimental section. Isoniazid, nowadays a widely used anti-tubercular drug recommended by the WHO [28,29], was used as positive control. 

The first evaluated compounds were the natural ones, compounds **1** and **2**, to provide standards for evaluation and goals in the search for more active compounds. Both compounds gave a MIC value of 125 μg·mL^−1^, which is unpromising. According to Cantrell [30], only substances with MIC values equal or less than 64 μg·mL^−1^ are considered to be active against mycobacteria. The same author classifies substances with MIC values equal or higher than 128 μg·mL^−1^ as inactive, and describes substances with MIC values between 64 and 32 μg·mL^−1^ as moderately active [30]. We agree with Cantrell, and propose here a more detailed and complete scale for the classification of anti-mycobacterial activity, covering all ranges (Table 1). 

**Table 1:** Classification of compounds with anti-mycobacterial activities.

Activity Level	Range of MIC Values (μg·mL^−1^)
Inactive	125 and higher
Weak	80 to 100
Moderate	31.25 to 64
Significant	12.5 to 25
Promising	10 or lower

The values that define each range were chosen taking into account the different values used in different dilution values during different authors’ MIC measurements, seen in review papers that gather several values from different articles [30]. Some authors use values from 1000 to 0.49 μg·mL^−1^, while others prefer dilutions from 1600 to 0.78 μg·mL^−1^. To define the promising range, we also took into account the MIC values obtained for the first line anti-tubercular drugs (Table 2). As mentioned, our main goal was to reach moderate to significant activities with the modifications, since we are starting from inactive substances (with MIC of 125 μg·mL^−1^).

**Table 2:** Results obtained in the anti-tuberculosis potential and toxicity evaluation together with reference values for first line drugs.

Compound	Obtention		MIC (μg·mL^−1^)	MIC (μM)	Toxicity ** (Cell Viability)
	Origin Compound	Process			
1	-----	Isolation	125	---	ne
2	-----	Isolation	125	---	ne
3	1	Epoxidation	100	---	ne
4	1	Ozonolisis	100	---	ne
5	4	Baeyer-Villiger	200	---	ne
6	2	Epoxidation	25	78	97%
7	2	Ozonolisis	12.5	47.3	96.7%
8	7	Aldol reaction	6.25	25.36	100%
Ethambutol * [31]	-----	---	1.64	7.22	---
Pyrazinamide [32]	-----	---	3.12	25.34	---
Streptomycin [32]	-----	---	6.25	10.75	---
Rifampicin [32]	-----	---	0.12	0.15	---
Isoniazid	-----	---	0.06	0.44	---

* Ethambutol dihydrochloride (EMB^.^2HCl); ** Evaluated at the active concentration; ne = not evaluated.

The kaurenoic acid derivatives achieved a part of our objectives, due to the obtainment of lower MIC values (see Table 2). Nevertheless, the best results obtained with kaurenoic acid derivatives were MIC values of 100 μg·mL^−1^, which can only be considered weak activity. This is still far from the significant or promising range. Moreover, one of the obtained derivatives (compound **5**) presented an even higher MIC than the natural product. 

On the other hand, copalic acid derivatives led us to reach our objectives and achieve even better results, presenting considerable improvement in activity. All the copalic acid derivatives presented MIC values lower than the kaurenoic acid derivatives. The epoxidation product, compound **6**, reached the level of significant activity with a MIC value of 25 μg·mL^−1^, one fifth of the original value (125 μg·mL^−1^ for the natural product). An even better result was obtained with compound **7**, which presented a MIC value of 12.5 μg·mL^−1^, virtually placing this compound in the promising range.

The most important result of our work was the achievement of a derivative with the same activity as a first line drug. Compound **8** presented a MIC value of 6.25 μg·mL^−1^, the same value as found for streptomycin [32]. This is an important result, since it is the same activity as that presented by a first line drug currently in use for anti-tuberculosis treatment (see Table 2). 

Further considerations lead to other interesting insights. When a comparison between compound **8** and streptomycin is accomplished with MIC values in micromolar concentrations, streptomycin seems to be better (10.75 μM) than compound **8** (25.36 μM). Nevertheless, this MIC value in micromolar concentrations is equivalent to pyrazinamide (25.34 μM) [32], another first line drug, also currently in use in the treatment of TB. In this case, the synthetic derivative (**8**) equaled the activity of a substance that seemed to be substantially better (with MIC value of 3.12 μg·mL^−1^). Thus, compound **8** equalled the anti-tubercular activity of two first line anti-TB drugs, according to the observed MIC values.

One also has to take into account the toxicity of these anti-TB agents. The three compounds with better results for anti-tubercular activity were assayed to evaluate their toxicity. The results are shown in Table 2. Compound **8** was evaluated for its cytotoxicity and showed a result of 100% cell viability in the concentration of 7.8 μg·mL^−1^. This indicates that this substance is not toxic at the active concentration. 

## 3. Experimental Section 

### 3.1. General Methods

Unless stated otherwise all reagents were purchased from either Sigma-Aldrich (Gillingham, UK) or Alfa-Aesar (Heysham, Lancashire, UK). *m-*CPBA (57%–86%; Attention! potentially explosive) was purified on a 35 g scale by dissolving in 250 mL diethyl ether and washed against saturated sodium carbonate solution (3 × 150 mL). The remaining ether was dried (under MgSO_4_), and remaining solvents were removed under reduced pressure affording 17 g of pure *m*-CPBA [33]. Non-aqueous solvents used were pre-dried before use, by distillation under nitrogen over the appropriate drying reagent: tetrahydrofuran and petroleum ether (40/60) was distilled over sodium benzophenone ketyl; dichloromethane was distilled over calcium hydride. All reactions were performed under an atmosphere of nitrogen, with the round bottom flask pre-dried under a flow of nitrogen whilst heating with a flame-gun.

### 3.2. General Methods for Substance Analysis

^1^H- and ^13^C-NMR spectra were recorded using a Bruker Avance III spectrometer (operating frequency 500.21 MHz for ^1^H and 125.05 MHz for ^13^C). Chemical shift values are quoted in parts per million (ppm, *δ*), and coupling constants *J* are quoted in Hertz (Hz). High resolution mass spectra (HRMS) were obtained from the service provided by the EPSRC Mass Spectrometry Service at the University of Swansea. HRMS were run on an LTQ Orbitrap XL instrument as solid samples utilizing an ASAP probe. Data were collected using APCl (ionization mode) and FTMS (FT analyzer). Infrared spectra were acquired using a PerkinElmer spectrophotometer. Solid samples and liquid samples were run neat utilizing the ATR unit of the spectrometer.

### 3.3. Anti-Tuberculosis Activity Assay

The study of antimycobacterial activity of the derivatives obtained was performed in LAPEMA (Research Laboratory of Applied Microbiology)—UNIFRAN, under the responsibility of Carlos Henrique Gomes Martins, developer for the tests. The activity was determined by the minimum inhibitory concentration (MIC). The MIC is defined as the lowest concentration of antimicrobial agent that completely inhibits bacterial growth. The MIC values were determined in triplicate using the microdilution technique on a REMA, adapted from a procedure reported in the literature [34]. 

One milligram of each diterpene was dissolved in 125 μL of dimethylsulfoxide (DMSO) and 1875 μL of Mueller Hinton broth was added. The final concentration of DMSO did not exceed 5% and the percentage of this solution was used as negative control. With the aid of a sterilized platinum loop, the 24-hour cultures of microorganisms were taken and transferred to tubes containing 10 mL of sterile saline solution. The suspension was standardized by comparing it with the McFarland tube 0.5 (0.1 mL of a 1% solution of BaCl_2_ in 9.9 mL of a 1% H_2_SO_4_). Then, serial dilutions in saline and finally in Mueller Hinton were performed to provide an inoculum of 5 × 105 CFU/mL (colony forming unit/mL).

Microplates with 96 holes were sterilized, and a total of 100 μL Mueller Hinton was added, with suspensions of micro-organisms and the solutions of the metabolites to be evaluated. Diterpenes were evaluated at different concentrations allowing one to determine the concentration required to inhibit the growth of micro-organisms to be evaluated. In one of the holes of each plate was made the control culture, which must provide bacterial growth due to the absence of antimicrobial agents. Another hole was used for sterility control of the medium and Mueller Hinton, and another one for solvent control (DMSO) used to solubilize the diterpenes. As a positive control, it was used isoniazid.

The plates were incubated at 37 °C for 24 h. Subsequently, to each well it was added 30 μL of 0.02% aqueous solution of resazurin. After waiting for 18 h, the presence of blue coloration (coloring resazurin solution) was interpreted as lack of bacterial growth and pink color signifies the presence of viable micro-organisms. 

### 3.4. Natural Products Extraction

#### 3.4.1. Kaurenoic Acid

A portion (1 kg) from a commercial sample of the dried and ground aerial parts of *Mikania glomerata* was extracted portion wise in beaker with dichloromethane under ultrasound. The mixture was then filtered and the solvent was removed by rotary evaporation under reduced pressure. The solvent was recovered from the process and reused. This portion of plant yielded 42 g of crude extract, which was suspended in 300 mL of a 9:1 mixture of MeOH/H_2_O (*v*/*v*) and filtered through filter paper. The soluble portion was partitioned with *n*-hexane (4 × 300 mL) and dichloromethane (2 × 300 mL) and both fractions had their solvent removed by rotary evaporation. The *n*-hexane fraction, containing most of **1** was subjected to vacuum liquid chromatography (VLC) with *n*-hexane/EtOAc mixtures with increasing gradient of polarity, thereby obtaining 12 fractions. Fractions with large quantities of **1** were purified by open classic column chromatography (CCC) (*n*-hexane/EtOAc 9:1), resulting in 900 mg of kaurenoic acid.

#### 3.4.2. Copalic Acid

A sample (30 g) of the purchased *C. langsdorffii* oilresin was incorporated to 60 g of silica gel 60 and submitted to a vacuum liquid chromatography (VLC) with 380 g of silica gel (60 and 60H, 50% each), according to the adaptation method [35]. As eluent were used *n*-hexane/EtOAc mixtures with increasing gradient of polarity, thereby obtaining 7 fractions. Fractions with great quantities of **2** were purified by classic column chromatography (CCC) (*n*-hexane/EtOAc 9:1), resulting in 5.5 g of copalic acid (**2**).

### 3.5. Individual Experiments

*(2R,4'R,4a'S,6a'S,9'R,11a'R,11b'S)-4',11b'-Dimethyldodecahydro-7'H-spiro[oxirane-2,8'-[6a,9]methanocyclohepta[a]naphthalene]-4'-carboxylic acid* (**3**): A sample of kaurenoic acid (1, 0.1 g, 0.33 mmol) and chloroform (10 mL) were added to a 50 mL test tube, and the solution was stirred and then cooled to 0 °C. Whilst the temperature was maintained at 0 °C, purified *m*-CPBA (0.11 g, 0.66 mmol) was added in one portion, and the reaction was kept stirring for 24 h at 0 °C. The reaction was quenched by the addition of saturated sodium hydrogen carbonate solution (20 mL). The organic layer was separated, and was subsequently submitted to consecutive washings with saturated sodium hydrogen carbonate solution (5 × 20 mL). The organic layer was dried over MgSO_4_, and the remaining solvents were removed under reduced pressure, affording the title compound **3** (0.094 g, 90%) as a pure colourless oil without any need for further purification. ATR-FTIR *ν*_max_/cm^−1^: 2936, 2253, 1691, 1460, 1265, 910. ^1^H-NMR (CDCl_3_, 500 MHz, δ ppm): 2.82 (d, 1H, *J* = 4.7 Hz), 2.74 (d, 1H, *J* = 4.7 Hz), 2.14–2.09 (m, 1H), 2.01–1.97 (m, 1H), 1.89–1.77 (m, 4H), 1.76–1.71 (m, 1H), 1.70–1.65 (m, 2H), 1.64–1.61 (m, 3H), 1.60–1.56 (m, 1H), 1.52–1.46 (m, 2H), 1.45–1.41 (m, 2H), 1.40–1.36 (m, 2H), 1.18 (s, 3H), 1.05–0.98 (m, 2H), 0.90 (s, 3H), 0.76 (m, 1H). ^13^C-NMR (CDCl_3_, 125 MHz, δ ppm): 183.7, 66.6, 57.1, 55.2, 50.6, 48.9, 45.6, 43.9, 42.7, 41.4, 40.9, 39.9, 38.7, 38.0, 29.23, 29.16, 22.0, 19.8, 19.3, 16.0. HRMS: [C_20_H_30_O_3_ − H]^−^ requires 317.2122; Found 317.2118 [M − H]^−^.

*(4R,4aS,6aS,9R,11aR,11bS)-4,11b-Dimethyl-8-oxotetradecahydro-6a,9-methanocyclohepta[a]naphthalene-4-carboxylic acid* (**4**): To a pre-dried 100 mL round bottom flask, kaurenoic acid **1** (0.1 g, 0.33 mmol) and anhydrous dichloromethane (20 mL) were added. Under stirring, the solution was cooled to −72 °C (dry ice/ethanol) and, after 30 min, a stream of oxygen/ozone was passed through the solution maintained at −72 °C. Then, a steady stream of ozone (O_3_) was bubbled through this solution (10 min) until the solution became pale blue in colour. Gaseous oxygen was then purged into the system to remove ozone excess. Dimethylsulfide was then added to the resulting mixture at −72 °C, and the mixture was kept stirring for 30 min, while it was warmed to room temperature. Saturated sodium hydrogen carbonate solution (50 mL) was then added to the reaction mixture, and the aqueous layer was extracted with dichloromethane (3 × 50 mL). The combined organic extracts were dried over MgSO_4_, and then remaining organic solvents were removed under reduced pressure. Purification was achieved by flash column chromatography on silica gel using petroleum ether (40/60): ethyl acetate (3:2) as the eluent, affording **4** as colourless oil (0.085 g, 85%). ATR-FT IR *ν*_max_/cm^−1^: 2942, 2871, 2252, 1738, 1694, 1399, 732. ^1^H-NMR (CDCl_3_, 500 MHz, δ ppm): 2.34 (dd, 1H, *J* = 4.3;7.3 Hz), 2.25 (dd, 1H, *J* = 2.9;12.1 Hz), 2.15–2.09 (m, 1H), 1.95–1.88 (m, 2H), 1.87–1.80 (m, 4H), 1.79–1.67 (m, 3H), 1.65–1.56 (m, 2H), 1.55–1.46 (m, 2H), 1.44–1.36 (m, 2H), 1.20 (s, 3H), 1.12 (d, 1H, *J* = 8.1 Hz), 1.05 (dd, 1H, *J* = 2.8; 11.5 Hz), 0.97 (dd, 1H, *J* = 4.7;13.6 Hz), 0.95 (s, 3H), 0.83–0.73 (m, 1H). ^13^C-NMR (CDCl_3_, 125 MHz, δ ppm): 222.8, 184.1, 56.8, 55.0, 54.0, 47.8, 43.7, 42.5, 41.0, 40.6, 39.8, 37.7, 37.3, 29.5, 29.0, 20.7, 19.0, 18.8, 16.1. HRMS: [C_19_H_28_O_3_ − H]^−^ requires 303.1966; Found 303.1959 [M − H]^−^.

*(3R,6aS,8aS,9R,12aS,12bR)-9,12a-Dimethyl-5-oxotetradecahydro-3,6a-methanonaphtho[2,1-d]oxocine-9-carboxylic acid* (**5**): The ozonolysis product, compound **4** (0.1 g, 0.33 mmol), was added to a test tube together with dichloromethane (10 mL) at room temperature. To this solution, a sample of *m*-CPBA (0.1 g, 0.66 mmol) was added, and it was kept under stirring at room temperature for 20 h. The reaction progress was monitored by thin layer chromatography, and after completion it was quenched using saturated sodium hydrogen carbonate solution (20 mL). The aqueous layer was extracted with dichloromethane (3 × 50 mL). The organic layers were combined, and then dried over magnesium sulphate followed by removal of remaining organic solvents under reduced pressure. The remaining residue was purified by open column chromatography on silica gel using petroleum ether: ethyl acetate as the eluent in a ratio of 3:2, affording **5** as colourless oil (0.097 g, 92%). ATR-FT IR *ν*_max_/cm^−1^: 2955, 2873, 2851, 1724, 1692, 1457, 1388, 1237, 1162. ^1^H-NMR (CDCl_3_, 500 MHz, δ ppm): 4.72 (m, 1H), 2.25 (dd, 1H, *J* = 2.2;18.6 Hz), 2.19–2.12 (m, 2H), 1.91–1.81 (m, 2H), 1.80–1.74 (m, 3H), 1.73–1.63 (m, 2H), 1.59–1.52 (m, 1H), 1.51–1.47 (m, 2H), 1.47–1.38 (m, 2H), 1.25–1.16 (m, 2H), 1.20 (s, 3H), 1.06–1.01 (m, 2H), 1.00–0.92 (m, 1H), 0.90 (s, 3H), 0.87–0.78 (m, 1H). ^13^C-NMR (CDCl_3_, 125 MHz, δ ppm): 183.4, 172.5, 76.1, 56.9, 53.4, 48.3, 43.8, 43.2, 41.3, 39.8, 37.7, 34.1, 33.4, 29.2, 29.0, 20.1, 19.3, 16.63, 16.58. HRMS: [C_19_H_28_O_4_ − H]^−^ requires 319.1915. Found 319.1913 [M − H]^−^.

*(E)-3-Methyl-5-((1S,2S,4aR,8aR)-5,5,8a-trimethyloctahydro-1H-spiro[naphthalene-2,2'-oxiran]-1-yl)pent-2-enoic acid* (**6**): To a test tube it was added copalic acid (**2**, 0.1 g, 0.33 mmol) and chloroform (10 mL). This solution was cooled to 0 °C and it was added *m*-CPBA (0.085 g, 0.5 mmol) to it in one portion, under stirring. The solution was kept under stirring for 24 h at 0 °C. The reaction was quenched by the addition of saturated sodium hydrogen carbonate solution (20 mL). The organic layer was submitted to consecutive washings with saturated sodium hydrogen carbonate solution (5 × 20 mL). It was then dried over MgSO_4_, and the remaining solvents were removed under reduced pressure, affording the title compound as pure colourless oil after purification by open column chromatography using petroleum ether: ethyl acetate (3:2) as the eluent (0.088 g, 87%). ATR-FT IR *ν*_max_/cm^−1^; 2944, 2868, 1694, 1640, 1575, 1436, 1255. ^1^H-NMR (CDCl_3_, 500 MHz, δ ppm): 5.60 (m, 1H), 2.68 (dd, 1H, *J* = 1.9;4.3 Hz), 2.44 (d, 1H, *J* = 4.3 Hz), 2.24–2.16 (m, 1H), 2.13–2.08 (m, 1H), 2.06 (d, 1H, *J* = 1.2 Hz), 1.87–1.79 (m, 1H), 1.79–1.71 (m, 2H), 1.67– 1.61 (m, 1H), 1.53–1.45 (m, 2H), 1.44–1.37 (m, 3H), 1.19 (t, 2H, *J* = 7.2 Hz), 1.16–1.07 (m, 2H), 1.00–0.93 (m, 2H), 0.83 (s, 3H), 0.82–0.77 (m, 2H), 0.76 (s, 3H), 0.74 (s, 3H). ^13^C-NMR (CDCl_3_, 125 MHz, δ ppm): 172.4, 163.9, 115.1, 59.2, 55.2, 53.5, 51.0, 43.0, 42.1, 40.6, 39.2, 36.6, 33.7, 33.6, 22.0, 21.8, 20.1, 19.5, 18.9, 14.8. HRMS: [C_20_H_32_O_3_ − H]^−^ requires 319.2279. Found 319.2273 [M − H]^−^.

*(1S,4aR,8aR)-5,5,8a-Trimethyl-1-(3-oxobutyl)octahydronaphthalen-2(1H)-one* (**7**): Copalic acid (**2**, 0.1 g, 0.33 mmol) and dry dichloromethane (20 mL) were added to a flame dried round bottom flask, and the stirring solution was cooled to −72 °C (dry ice/ethanol). After 30 min at this temperature, a stream of oxygen/ozone was passed through the solution at −72 °C. Then, a steady stream of ozone (O_3_) was bubbled through this solution (10 min) until the solution became pale blue in colour. Oxygen gas (O_2_) was then purged into the system to remove excess of ozone. Dimethylsulfide (0.2 mL) was then added to the resulting mixture at −72 °C, and the solution was allowed to stir and to warm to room temperature for 30 min. Saturated sodium hydrogen carbonate solution (50 mL) was then added to the reaction mixture, and the aqueous layer was extracted with dichloromethane (3 × 50 mL). The combined organic extracts were dried over MgSO_4_, and then the remaining organic solvents were removed under reduced pressure. Purification was achieved by flash column chromatography on silica gel using petroleum ether:ethyl acetate (3:2) as the eluent, affording **7** as a colourless oil (0.080 g, 92%). ATR-FT IR *ν*_max_/cm^−1^: 2949, 2869, 1711, 1444, 1164. ^1^H-NMR (CDCl_3_, 500 MHz, δ ppm): 2.56–2.49 (m, 1H), 2.34 (dd, 1H, *J* = 2.1;4.8 Hz), 2.25–2.17 (m, 1H), 2.12 (dt, 1H, *J* = 7.5;17.5 Hz), 2.09–2.04 (m, 1H), 2.03 (s, 3H), 1.77–1.64 (m, 2H), 1.60–1.51 (m, 2H), 1.53–1.47 (m, 2H), 1.46–1.39 (m, 2H), 1.38–1.31 (m, 1H), 1.20–1.09 (m, 2H), 0.89 (s, 3H), 0.78 (s, 3H), 0.66 (s, 3H). ^13^C-NMR (CDCl_3_, 125 MHz, δ ppm): 212.6, 209.5, 63.4, 54.4, 43.0, 42.9, 42.8, 42.0, 39.3, 33.9, 33.7, 30.1, 24.2, 22.0, 19.2, 16.3, 14.8. HRMS: [C_17_H_28_O_2_ + H]^+^ requires 265.2162. Found 265.2162 [M + H]^+^.

*(4aS,4bR,8aR,10aS)-10a-Hydroxy-4b,8,8-trimethyldodecahydrophenanthren-2(1H)-one* (**8**): To a 150 mL round bottom flask were added freshly distilled THF (10 mL) and potassium *tert*-butoxide (0.5 g, 4.46 mmol). At this point, a solution of copalic acid ozonolysis product **7** (0.1 g, 0.38 mmol), dissolved in distilled THF (5 mL), was added to the initial mixture. The mixture was kept under stirring overnight. Upon completion of the reaction (as observed by TLC), the solvent was removed under reduced pressure. The residue was dissolved in diethyl ether (10 mL) and washed with H_2_O acidified with a few drops of 1M HCl (2 × 5 mL). After the concentration of this new organic layer under reduced pressure, the remaining residue was purified by open silica gel column chromatography, using petroleum ether:ethyl acetate (4:1) as eluent. Compound **8** was isolated as colorless oil (0.083 g, 89%). ATR-FT IR *ν*_max_/cm^−1^: 2958, 2927, 2871, 1658, 1461, 1366. ^1^H-NMR (CDCl_3_, 500 MHz, δ ppm): 2.38 (m, 1H), 2.33 (dd, 1H, *J* = 0.7;14.2 Hz), 2.25–2.21 (m, 1H), 2.21–2.16 (dd, 1H, *J* = 2.5;14.2 Hz), 1.92–1.88 (m, 1H), 1.87–1.82 (m, 1H), 1.78–1.65 (m, 2H), 1.63–1.54 (m, 2H), 1.54–1.50 (m, 2H), 1.50–1.45 (m, 1H), 1.42–1.34 (m, 2H), 1.12 (m, 2H), 0.93 (s, 3H), 0.83 (s, 3H), 0.80 (s, 3H). ^13^C-NMR (CDCl_3,_ 125 MHz, δ ppm): 210.9, 75.8, 57.5, 56.3, 55.5, 42.4, 42.2, 41.7, 40.1, 37.7, 33.9, 33.5, 22.0, 21.7, 18.6, 18.2, 15.5. Due to its tertiary alcohol, compound **8** easily undergoes a dehydration reaction in the mass spectrometer’s ionizer. Thus, the observed mass is related to its dehydration product: HRMS: [C_17_H_26_O + H]^+^ requires 247.2056. Found 247.2055 [M + H]^+^.

### 3.6. Cytotoxic Assay

The cytotoxic activity was evaluated using the normal human fibroblast cell line GM07492-A (Coriell Cell Repositories, Camden, NJ, USA). Briefly, cells were trypsinized and seeded in 96-well plates at concentration of 4 × 10^4^ cells per well in DEMEN medium with HAM-F10 (1:1, *v*/*v*) (Sigma-Aldrich^®^, Saint Louis, MO, USA) supplemented with 10% fetal bovine serum (Life Technologies^®^, Grand Island, NY, USA). After 24 h of incubation at 37 °C (CO_2_ 5%), the cell cultures were treated with different concentrations of compounds 6, 7 and 8 dissolved in DMSO (1%) at concentrations ranging from 3.12 to 50 μg·mL^−1^ and incubated again for another 24 h. Then, the cell viability was assessed using the Cell Proliferation Kit II (Roche, Manheim, Germany) according to the manufacturer’s instructions. The cell viability was expressed as the percentage of negative control. Doxorubicin 2.17 μM was used as the positive control. The tests were performed in duplicate and repeated three times.

## 4. Conclusions 

We have shown that structural modifications to pre-existing natural products allow access to new analogues that, in turn, can exert a greater activity than the parent natural product when tested against tuberculosis cell lines. We have shown that structural modifications of copalic acid (**2**) through consecutive reactions, for example ozonolysis followed by aldol reaction, afforded highly biologically active compounds, such as **8**, that exhibit MIC values as low as 6.25 μg·mL^−1^. We have therefore achieved the synthesis of a non-toxic compound with an activity comparable to two of the first line drugs for tuberculosis treatment. 

## Supplementary Materials

Supplementary Materials are available and include ^1^H-NMR spectra, ^13^C-{^1^H}-NMR spectra and high resolution mass spectra for compounds **3**–**8**. Supplementary materials can be accessed at: http://www.mdpi.com1420-3049/20/10/18264/s1.
