# Supplementary material for: New Non-Toxic Semi-Synthetic Derivatives from Natural Diterpenes Displaying Anti-Tuberculosis Activity
Source: Molecules. 2015 Oct 7;20(10):18264–78. doi: 10.3390/molecules201018264 (PMC6331924; doi:10.3390/molecules201018264)
Supplement: Supplementary file 1 [file molecules-20-18264-s001.pdf]

# Supplementary Materials

## Contents


| | |
|---|---|
| Table of Contents | S1 |
| Compound **3** Spectra | S1/S2 |
| Compound **4** Spectra | S3/S4 |
| Compound **5** Spectra | S4/S5 |
| Compound **6** Spectra | S6/S7 |
| Compound **7** Spectra | S7/S8 |
| Compound **8** Spectra | S9/S10 |


## Compound 3

*$^1$H-NMR*

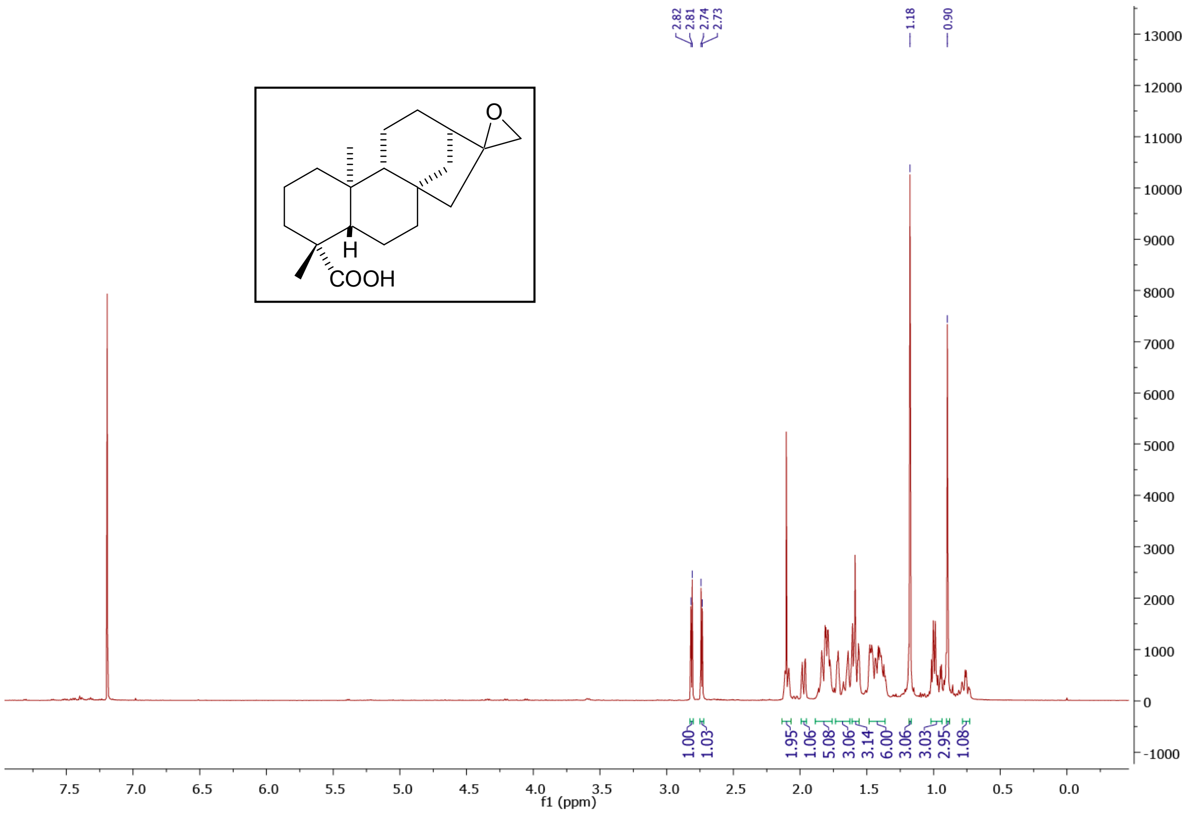

*$^{13}$C-NMR*

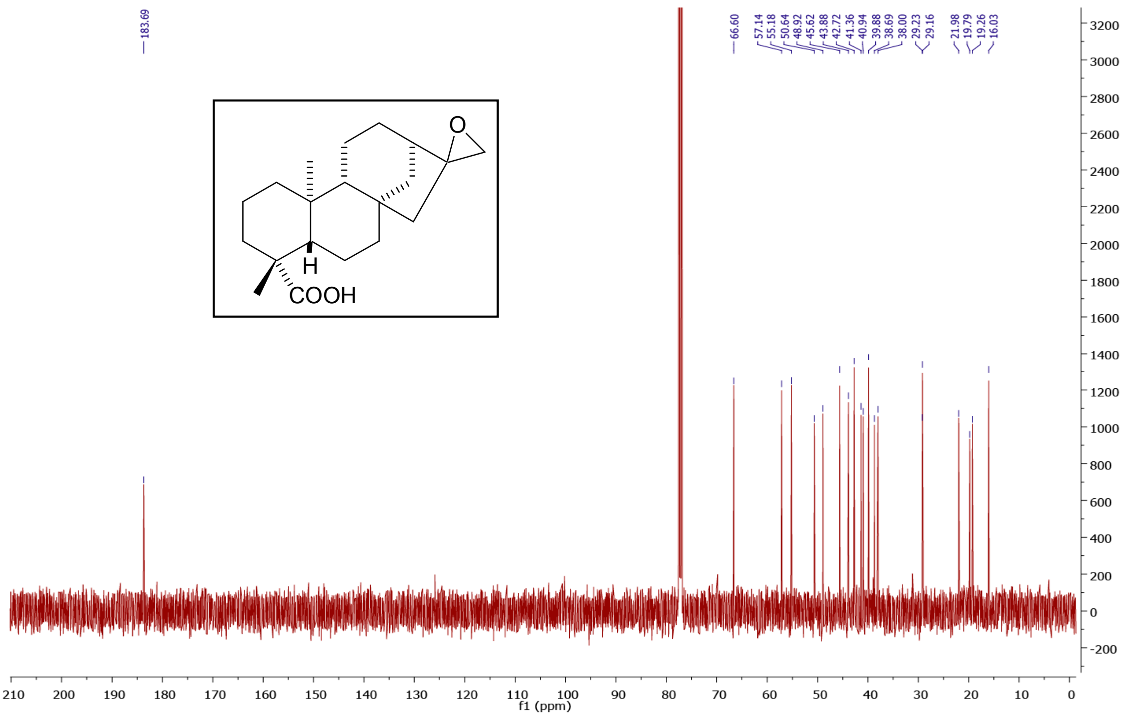
183.69
66.60
57.14
55.18
50.64
48.92
45.62
43.88
42.72
41.36
40.94
39.88
38.69
38.00
29.23
29.16
21.98
19.79
19.26
16.03
O
H
COOH
f1 (ppm)

*HRMS*

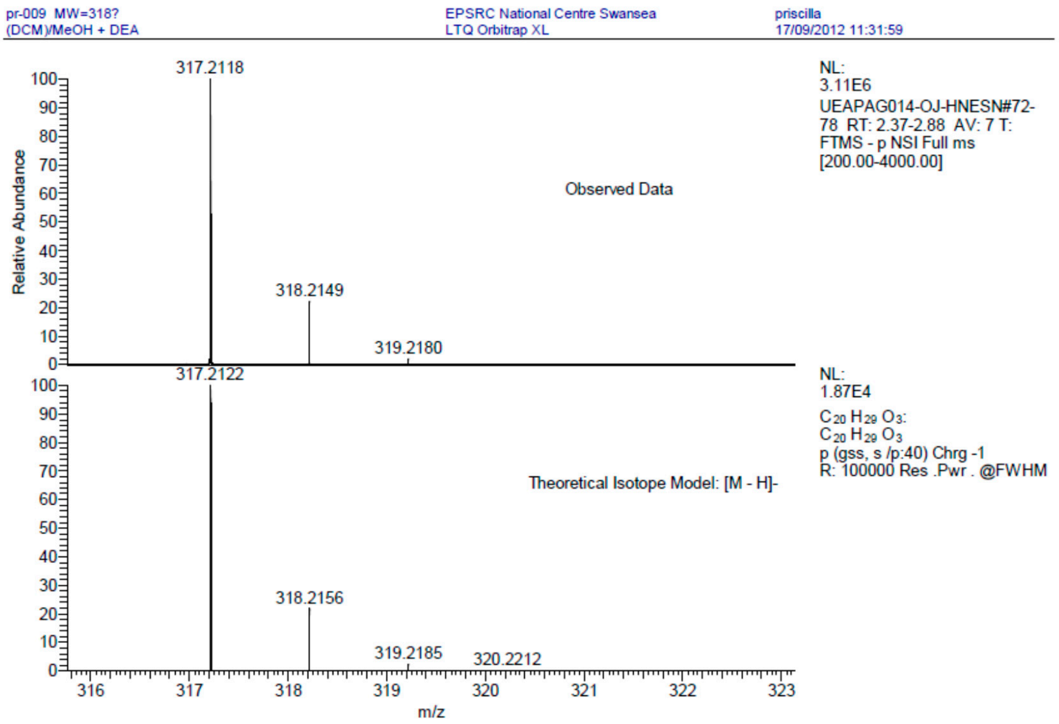
pr-009 MW=318?
(DCM)/MeOH + DEA
EPSRC National Centre Swansea
LTQ Orbitrap XL
priscilla
17/09/2012 11:31:59
317.2118
318.2149
319.2180
Observed Data
NL:
3.11E6
UEAPAG014-OJ-HNESN#72-78 RT: 2.37-2.88 AV: 7 T: FTMS - p NSI Full ms [200.00-4000.00]
317.2122
318.2156
319.2185
320.2212
Theoretical Isotope Model: [M - H]-
NL:
1.87E4
C20 H29 O3:
C20 H29 O3
p (gss, s /p:40) Chrg -1
R: 100000 Res .Pwr . @FWHM
Relative Abundance
m/z

## Compound 4

*¹H-NMR*

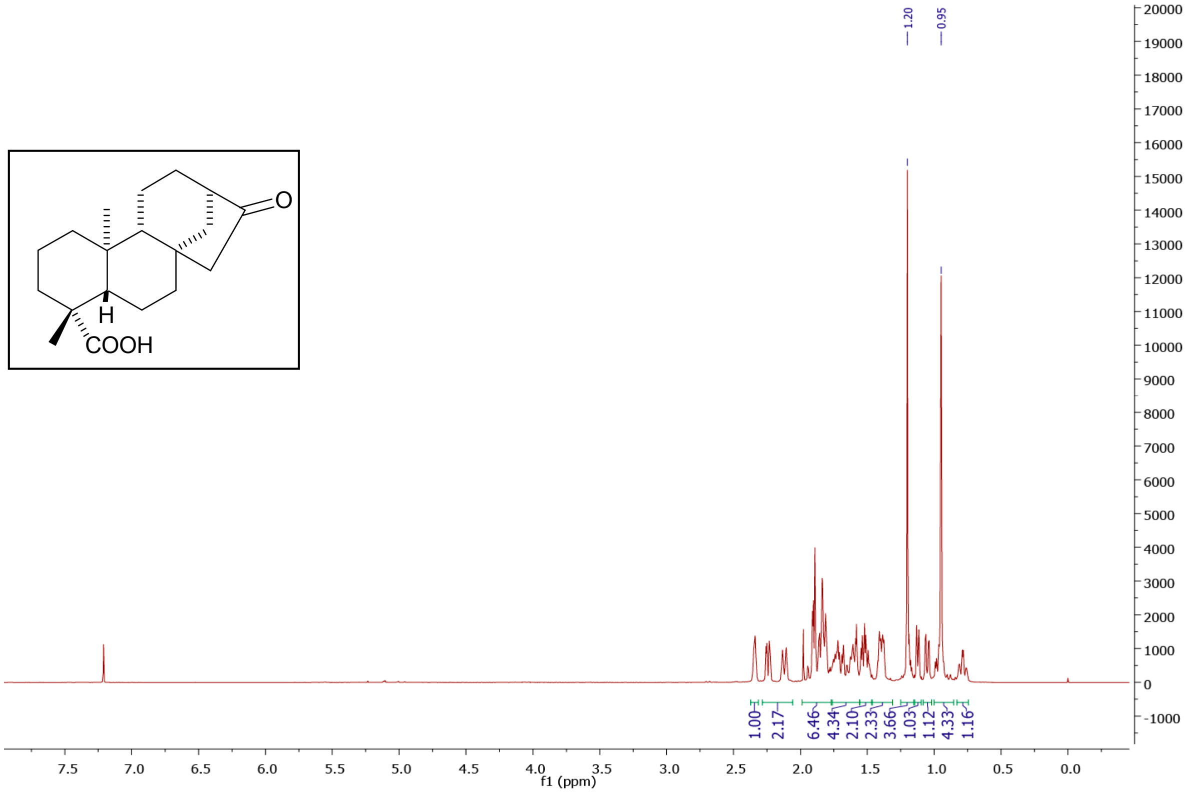


*¹³C-NMR*

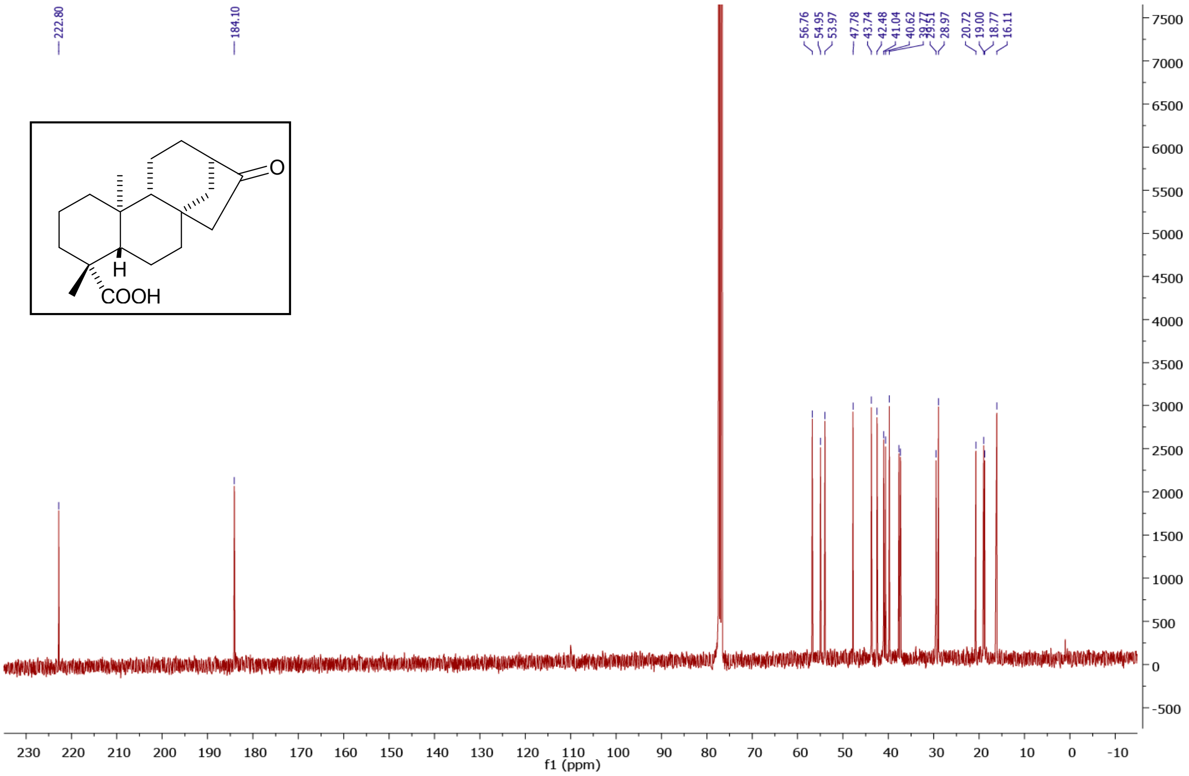

*HRMS*

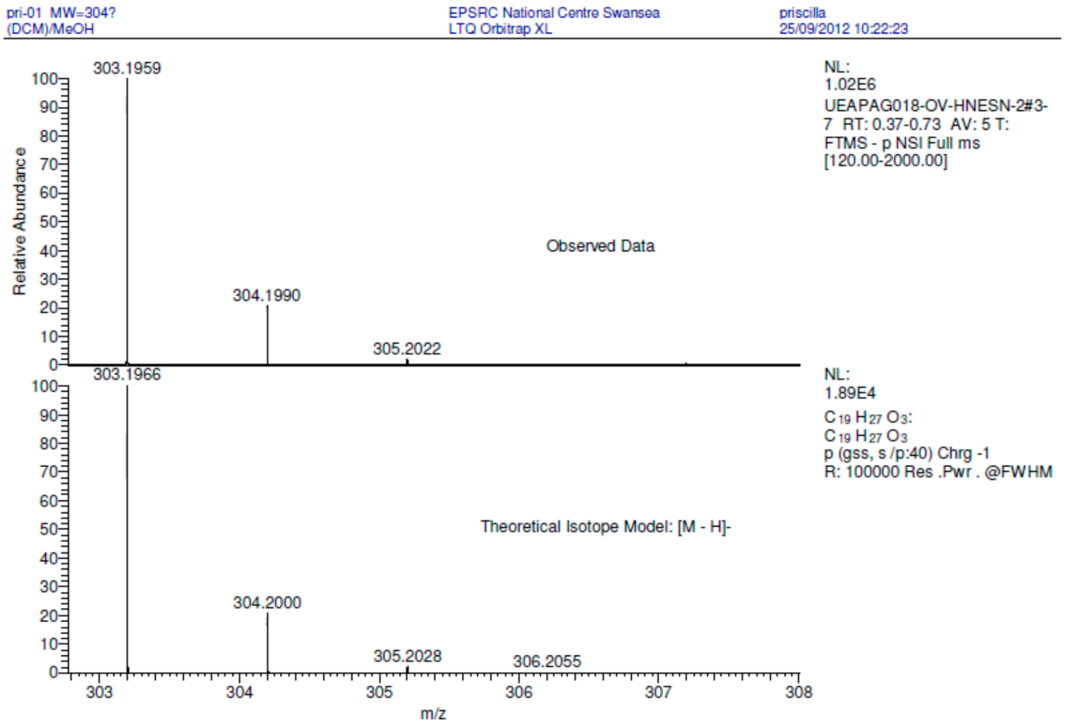


## Compound 5

*¹H-NMR*

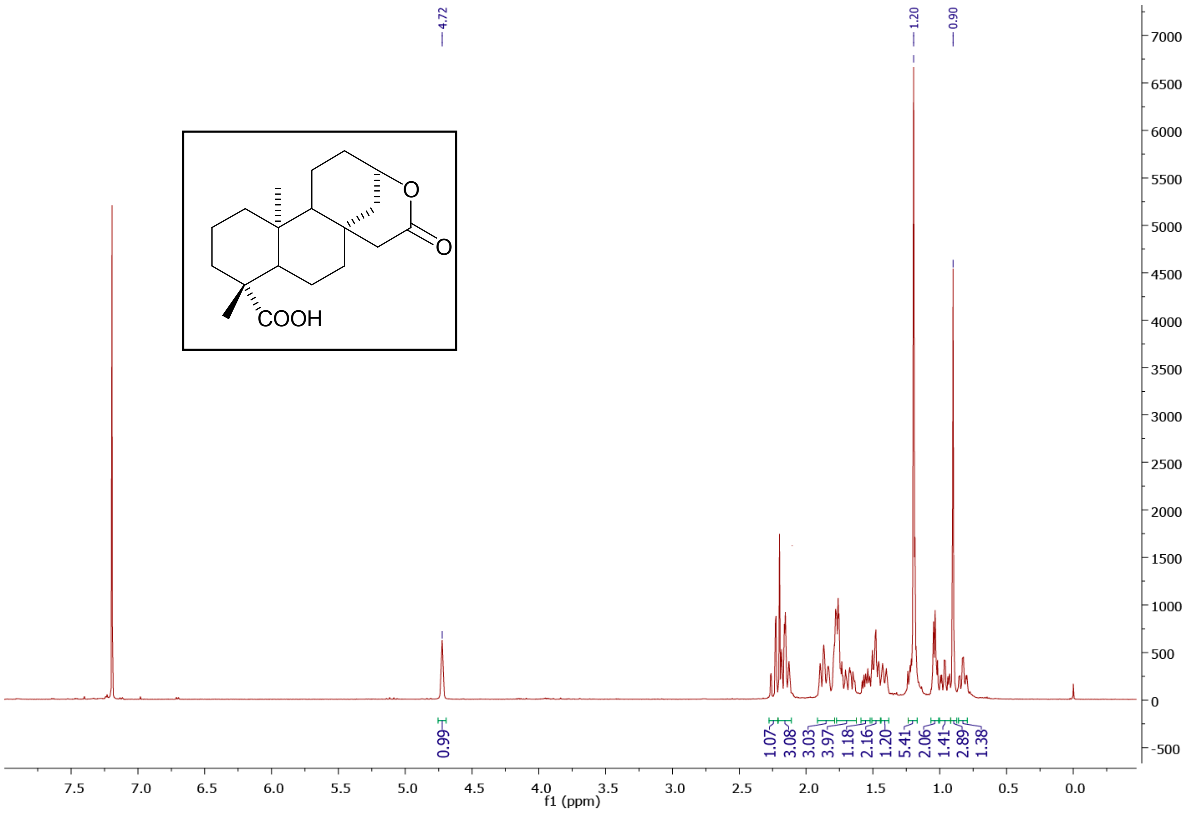

*$^{13}$C-NMR*

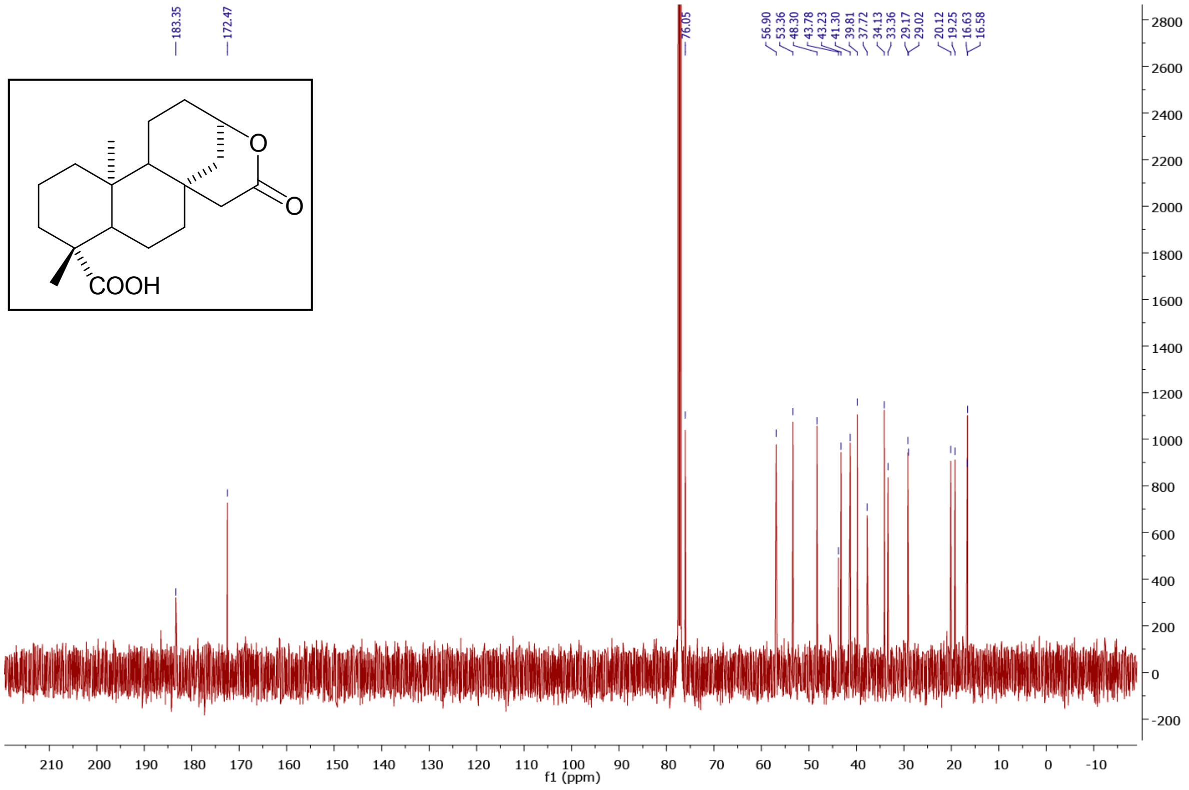
183.35
172.47
76.05
56.90
53.36
48.30
43.78
43.23
41.30
39.81
37.72
34.13
33.36
29.17
29.02
20.12
19.25
16.63
16.58
O
O
COOH
f1 (ppm)

*HRMS*

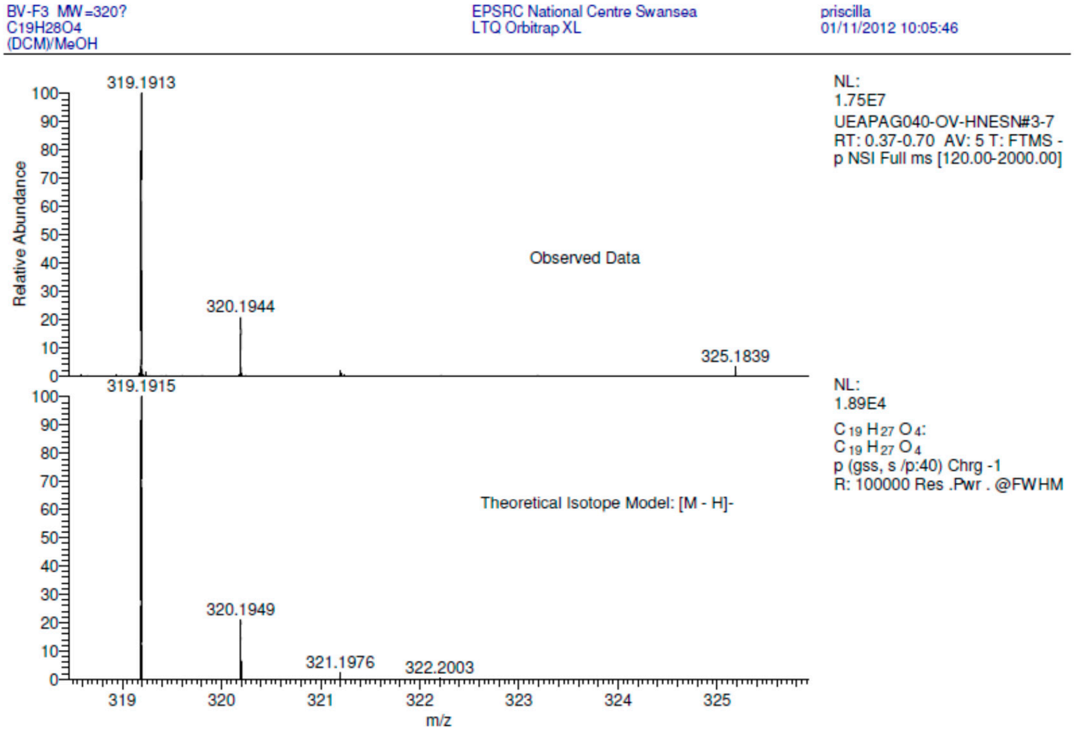
BV-F3 MW=320?
C19H28O4
(DCM)/MeOH
EPSRC National Centre Swansea
LTQ Orbitrap XL
priscilla
01/11/2012 10:05:46
319.1913
320.1944
325.1839
Observed Data
NL:
1.75E7
UEAPAG040-OV-HNESN#3-7
RT: 0.37-0.70 AV: 5 T: FTMS -
p NSI Full ms [120.00-2000.00]
Relative Abundance
319.1915
320.1949
321.1976
322.2003
Theoretical Isotope Model: [M - H]-
NL:
1.89E4
C 19 H 27 O 4:
C 19 H 27 O 4
p (gss, s /p:40) Chrg -1
R: 100000 Res .Pwr . @FWHM
m/z

## Compound 6

*¹H-NMR*

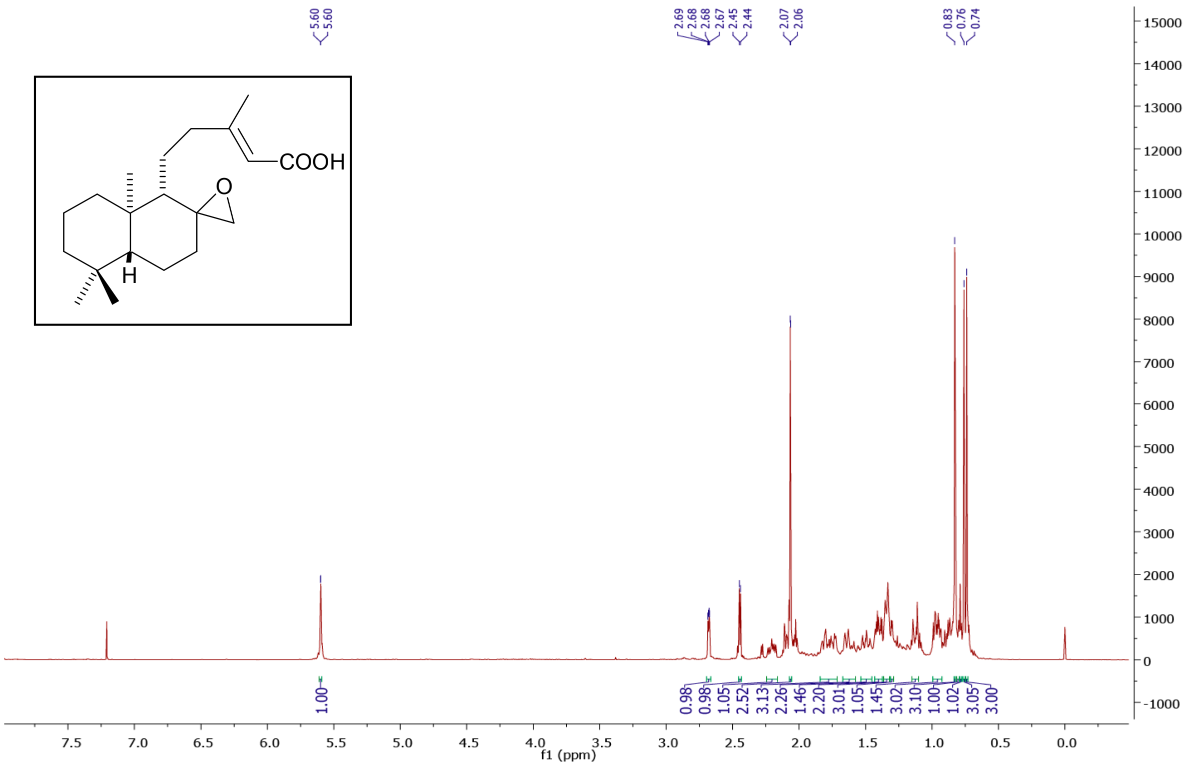

*¹³C-NMR*

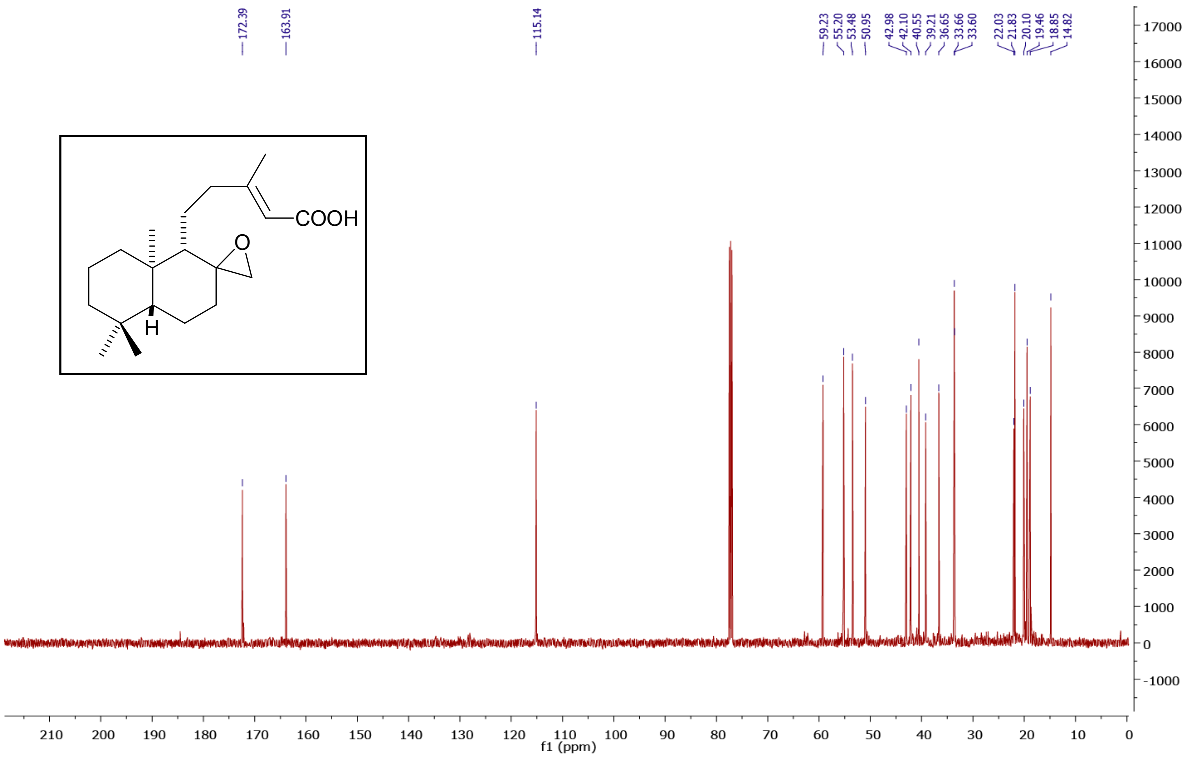

*HRMS*

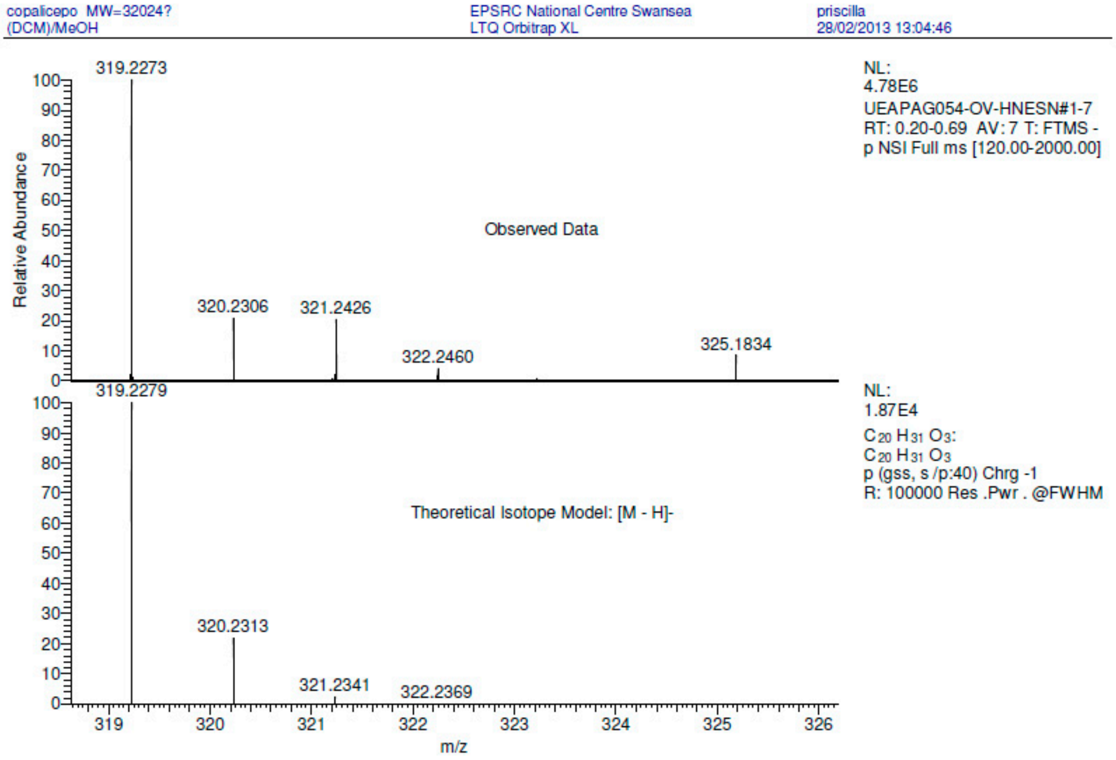


## Compound 7

*$^{1}$H-NMR*

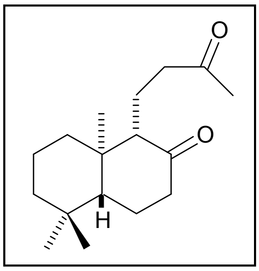


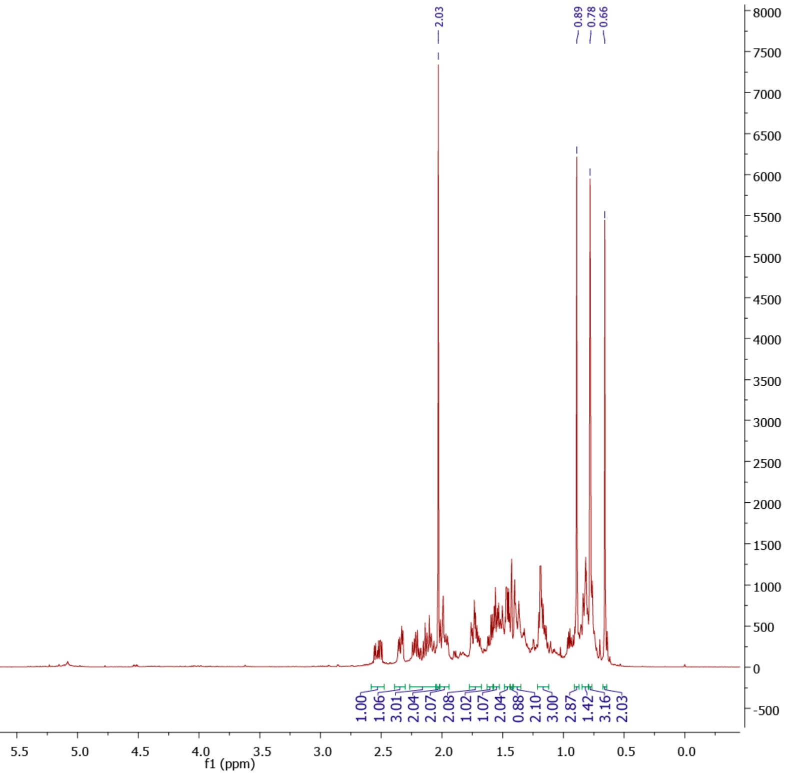

*$^{13}$C-NMR*

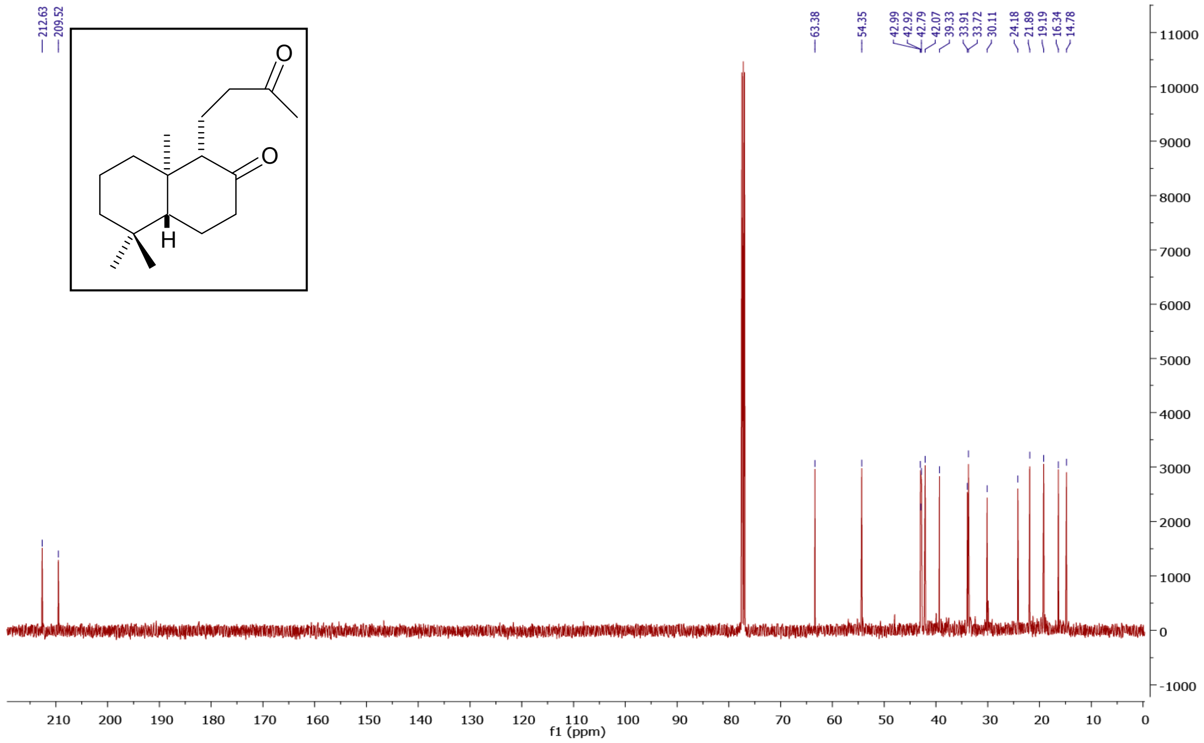
212.63
209.52
63.38
54.35
42.99
42.92
42.79
42.07
39.33
33.91
33.72
30.11
24.18
21.89
19.19
16.34
14.78
f1 (ppm)

*HRMS*

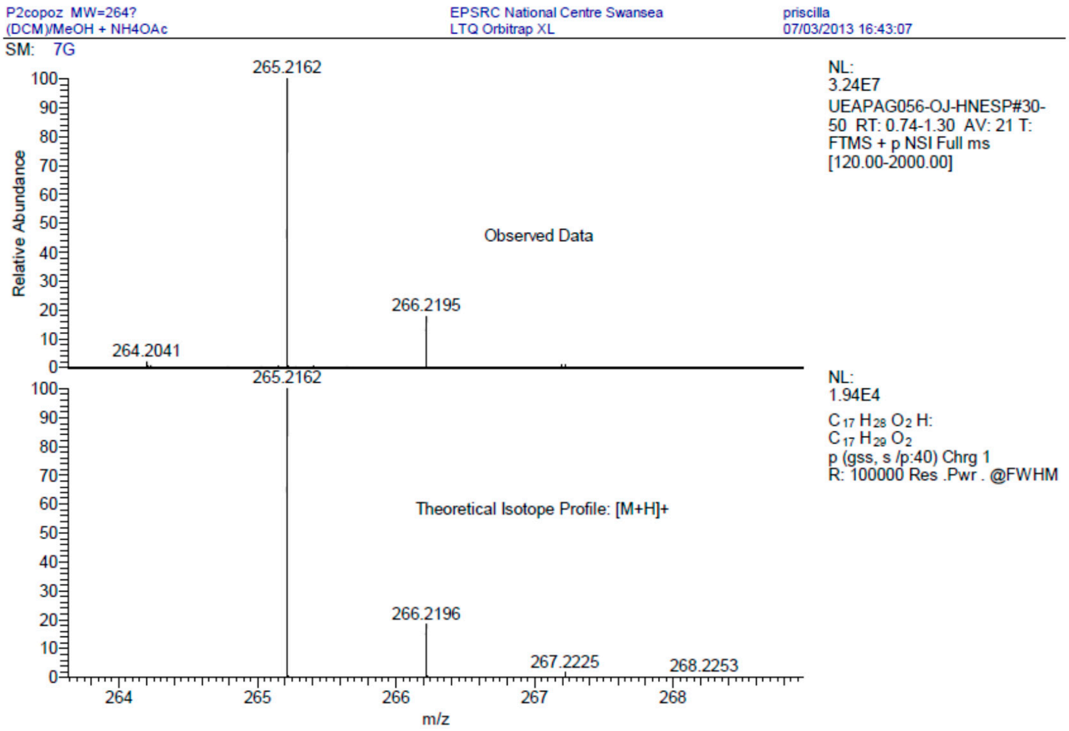
P2copoz MW=264?
(DCM)/MeOH + NH4OAc
EPSRC National Centre Swansea
LTQ Orbitrap XL
priscilla
07/03/2013 16:43:07
SM: 7G
265.2162
NL:
3.24E7
UEAPAG056-OJ-HNESP#30-50 RT: 0.74-1.30 AV: 21 T: FTMS + p NSI Full ms [120.00-2000.00]
Observed Data
266.2195
264.2041
Relative Abundance
265.2162
NL:
1.94E4
C17 H28 O2 H:
C17 H29 O2
p (gss, s /p:40) Chrg 1
R: 100000 Res .Pwr . @FWHM
Theoretical Isotope Profile: [M+H]+
266.2196
267.2225
268.2253
m/z

## Compound 8

*$^{1}$H-NMR*

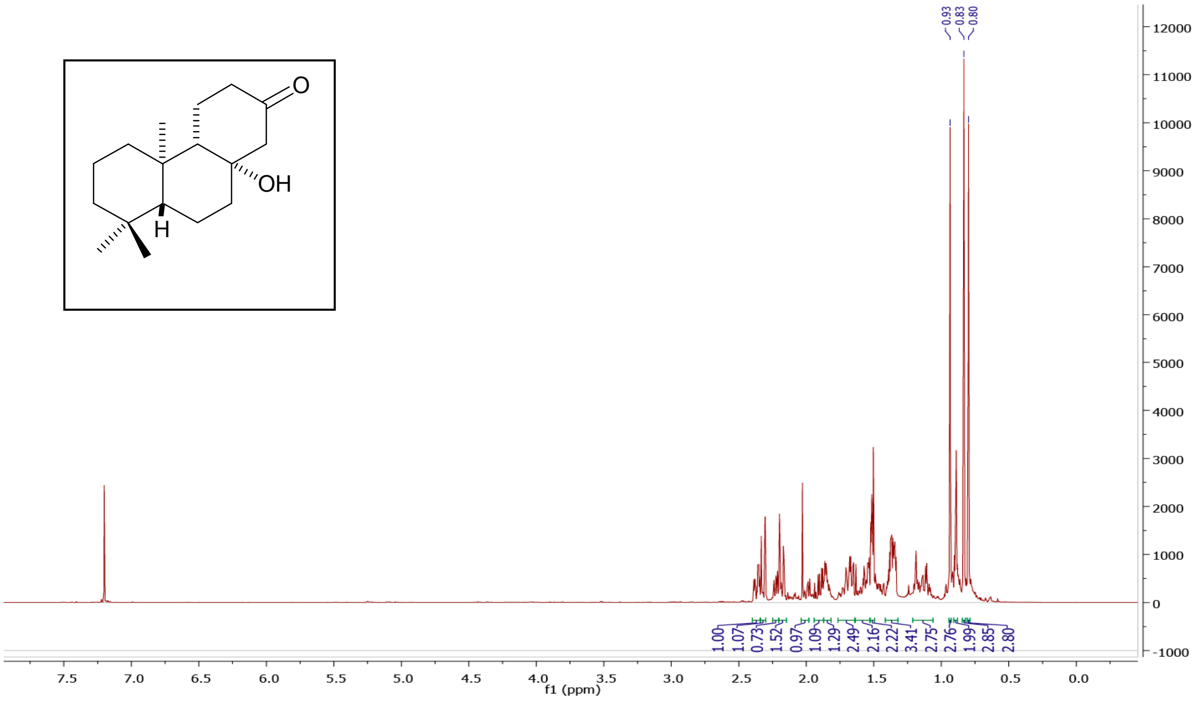


*$^{13}$C-NMR*

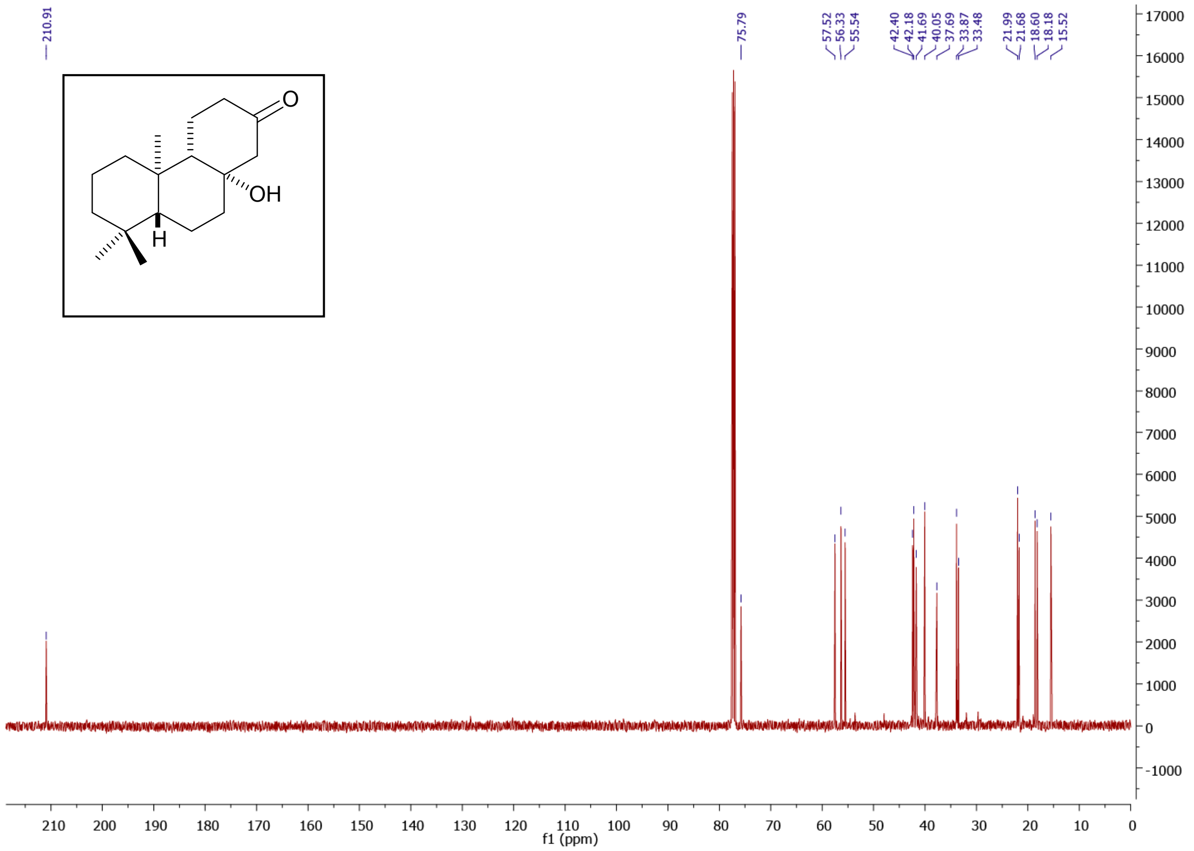

*HRMS*

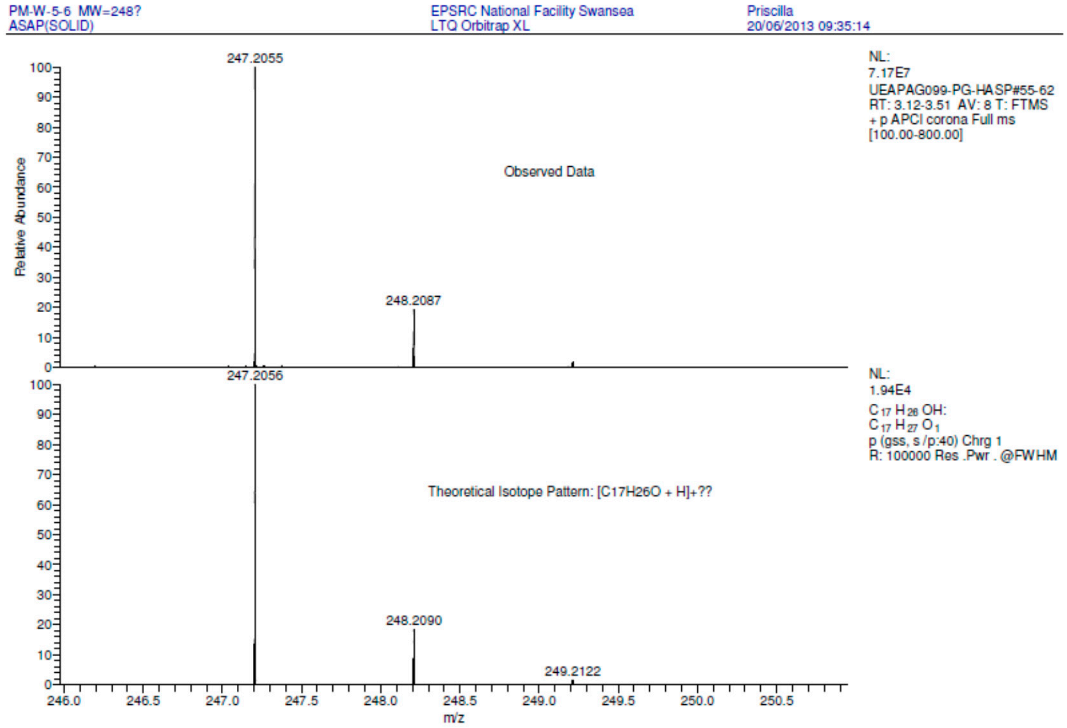
PM-W-5-6 MW=248?
ASAP(SOLID)
EPSRC National Facility Swansea
LTQ Orbitrap XL
Priscilla
20/06/2013 09:35:14
NL:
7.17E7
UEAPAG099-PG-HASP#55-62
RT: 3.12-3.51 AV: 8 T: FTMS
+ p APCI corona Full ms
[100.00-800.00]
247.2055
248.2087
Observed Data
Relative Abundance
NL:
1.94E4
C 17 H 26 OH:
C 17 H 27 O 1
p (gss, s /p:40) Chrg 1
R: 100000 Res .Pwr . @FWHM
247.2056
248.2090
249.2122
Theoretical Isotope Pattern: [C17H26O + H]+??
m/z
